# Supplementary material for: Splice-Junction-Based Mapping of Alternative Isoforms in the Human Proteome
Source: Cell Rep. Author manuscript; Available in PMC 2020 Jan 15. (PMC6961840; doi:10.1016/j.celrep.2019.11.026)
Supplement: 2 [file NIHMS1546469-supplement-2.zip › DF1/PXD010154/Esophagus/Esophagus_9_CNN1_YGVKPHDIFEANDLFENTNHTQVQSTLLALASMMG.pdf]

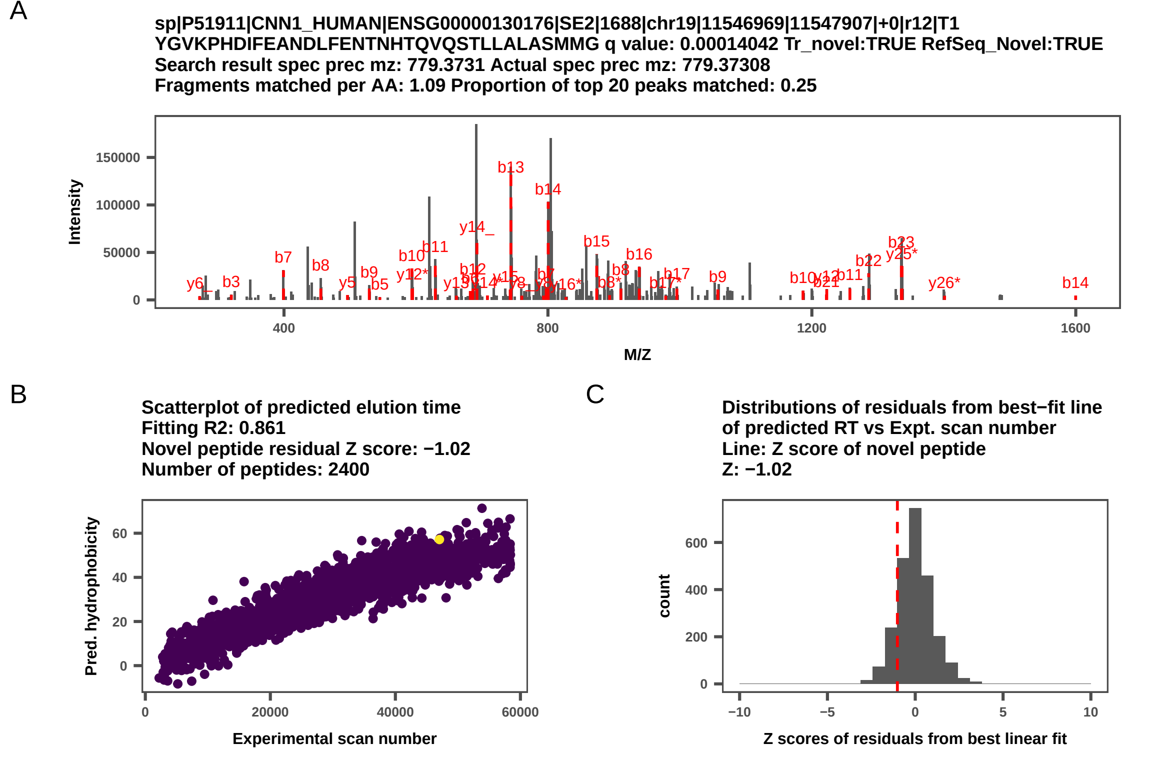

A
sp|P51911|CNN1_HUMAN|ENSG00000130176|SE2|1688|chr19|11546969|11547907|+0|r12|T1
YGVKPHDIFEANDLFENTNHTQVQSTLLALASMMG q value: 0.00014042 Tr_novel:TRUE RefSeq_Novel:TRUE
Search result spec prec mz: 779.3731 Actual spec prec mz: 779.37308
Fragments matched per AA: 1.09 Proportion of top 20 peaks matched: 0.25
Intensity
150000
100000
50000
0
400
800
1200
1600
M/Z
b3
b7
b8
y5
b9
b5
b10
b11
y12*
y14_
b12
b13
b14
b15
b16
b17
b9
b22
b23
y25*
y26*
b14
B
Scatterplot of predicted elution time
Fitting R2: 0.861
Novel peptide residual Z score: −1.02
Number of peptides: 2400
Pred. hydrophobicity
60
40
20
0
0
20000
40000
60000
Experimental scan number
C
Distributions of residuals from best−fit line
of predicted RT vs Expt. scan number
Line: Z score of novel peptide
Z: −1.02
count
600
400
200
0
−10
−5
0
5
10
Z scores of residuals from best linear fit
